# The Hippocampus Remains Activated over the Long Term for the Retrieval of Truly Episodic Memories

**DOI:** 10.1371/journal.pone.0043495

**Published:** 2012-08-24

**Authors:** Caroline Harand, Françoise Bertran, Renaud La Joie, Brigitte Landeau, Florence Mézenge, Béatrice Desgranges, Philippe Peigneux, Francis Eustache, Géraldine Rauchs

**Affiliations:** 1 INSERM, U1077, Caen, France; 2 Université de Caen Basse-Normandie, UMR-S1077, Caen, France; 3 Ecole Pratique des Hautes Etudes, UMR-S1077, Caen, France; 4 Centre Hospitalier Universitaire, U1077, Caen, France; 5 Service des Explorations Fonctionnelles Neurologiques, CHU, Caen, France; 6 Neuropsychology and Functional Neuroimaging Research Unit – UR2NF, Université Libre de Bruxelles, Bruxelles, Belgium; Wake Forest University, United States of America

## Abstract

The role of the hippocampus in declarative memory consolidation is a matter of intense debate. We investigated the neural substrates of memory retrieval for recent and remote information using functional magnetic resonance imaging (fMRI). 18 young, healthy participants learned a series of pictures. Then, during two fMRI recognition sessions, 3 days and 3 months later, they had to determine whether they recognized or not each picture using the “Remember/Know” procedure. Presentation of the same learned images at both delays allowed us to track the evolution of memories and distinguish consistently episodic memories from those that were initially episodic and then became familiar or semantic over time and were retrieved without any contextual detail. Hippocampal activation decreased over time for initially episodic, later semantic memories, but remained stable for consistently episodic ones, at least in its posterior part. For both types of memories, neocortical activations were observed at both delays, notably in the ventromedial prefrontal and anterior cingulate cortices. These activations may reflect a gradual reorganization of memory traces within neural networks. Our data indicate maintenance and strengthening of hippocampal and cortico-cortical connections in the consolidation and retrieval of episodic memories over time, in line with the Multiple Trace theory (Nadel and Moscovitch, 1997). At variance, memories becoming semantic over time consolidate through strengthening of cortico-cortical connections and progressive disengagement of the hippocampus.

## Introduction

One of the most amazing features of human memory is its capacity to retain memories on a time-scale ranging from milliseconds to the many decades that cover our life span [1]. The idea that memory traces are not immediately acquired in their definitive state but rather undergo a gradual process of consolidation over time dates back to observations made by the French psychologist Théodule Ribot in 1881. He was the first to report that, after brain damage, recently acquired information was more impaired than remote memories, a phenomenon now described as temporally graded amnesia or Ribot's law. Many years later, Cermak expounded the idea that episodic memory and semantic memory form a continuum in order to explain Ribot's gradient seen in amnesic syndrome [2], [3]. Accordingly, Ribot's temporal gradient would be due to the greater vulnerability to amnesia of episodic relative to semantic memory. In other words, the relatively better preservation of remote memory compared with recent memory in amnesic patients was linked to their semantic nature [4], [5].

Neuroscientists distinguish between two forms of memory consolidation. Synaptic consolidation is completed within minutes or hours that follow learning. It refers to a complex cascade of molecular and cellular events that are necessary to stabilize recently-acquired information within hippocampal networks. In contrast, system-level consolidation refers to a slow, time-dependent process that converts labile memory traces into more permanent and/or enhanced forms and implies the gradual reorganization of brain networks that support memory [6].

Studies conducted in brain-damaged patients, in animals and using functional neuroimaging techniques have provided remarkable insights on how remote and recent memory traces are reorganized and stored in the brain [7]. According to the standard model of memory consolidation [8], the medial temporal lobes, including the hippocampus, act as a temporary memory system strengthening cortico-cortical connections. With time, the contribution of the hippocampus gradually decreases with neocortical areas becoming able to support independently the retrieval of remote memories. However, the observation of patients with ungraded retrograde amnesia [7] challenged this view, eventually leading to the proposal of the Multiple Trace Theory which posits that the hippocampus plays a permanent role in memory storage and retrieval for episodic, but not for semantic memories [9]. This view also integrates the idea that the format of memory representations may considerably vary with the passage of time, for example by reducing the richness of details present during the initial encoding of the event and only retaining the more semantic aspects of the past.

**Figure 1:**
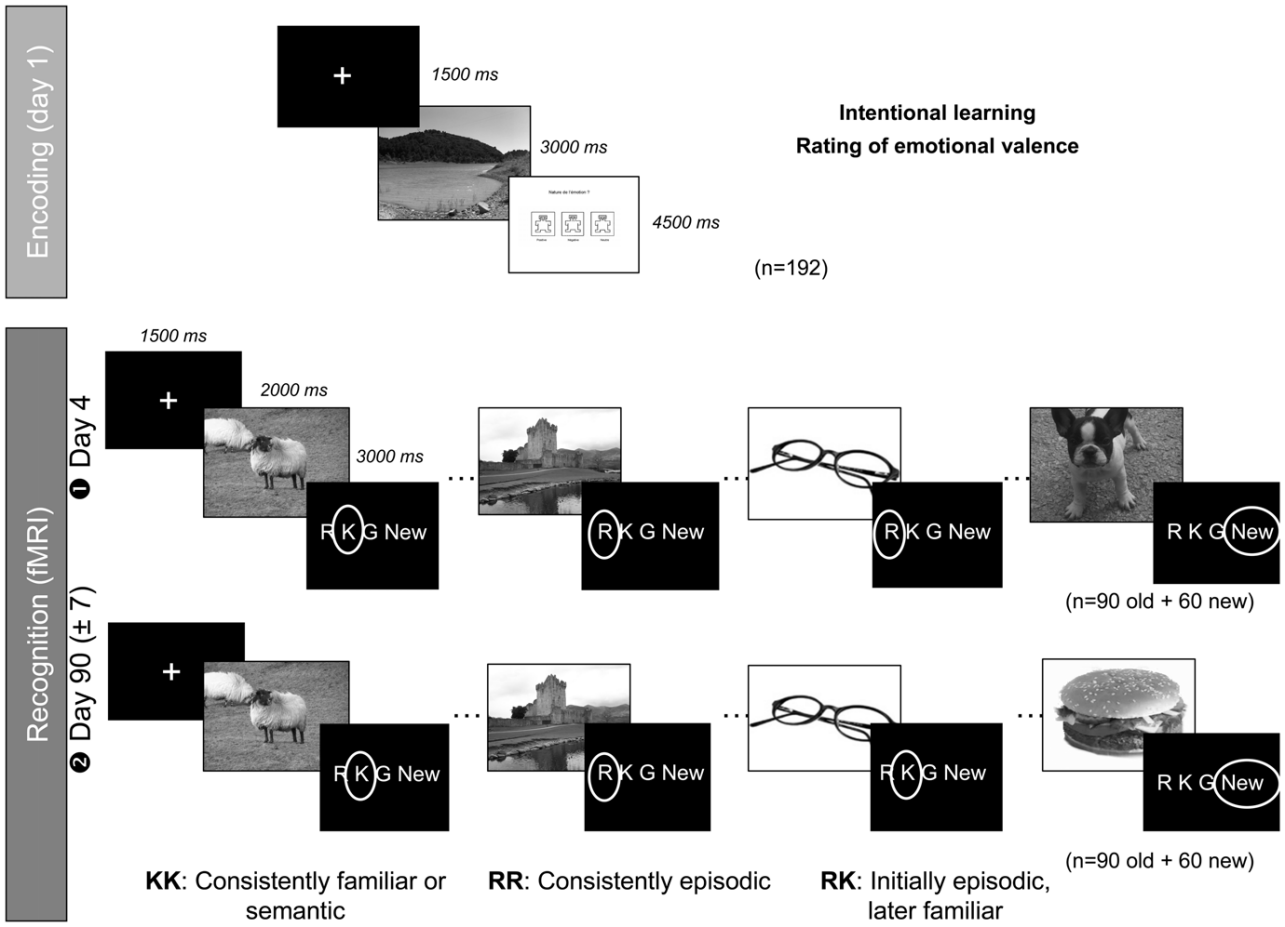
Experimental design. On day 1, participants were instructed to rate the valence of a series of emotional (positive and negative) and neutral colored pictures and to memorize them. Memory performance was tested on day 4 (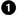) and day 90 (±7 days, 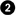) using a recognition task associated with the Remember/Know/Guess paradigm. Functional MRI data were acquired during these recognition sessions. After each session, all pictures that received an R response were presented again outside the scanner during a debriefing interview, to obtain from subjects a justification of their R responses with contextual details. Note that the same learned (“old” pictures) were used for both retrieval sessions allowing to track the fate of memories and their possible qualitative change over time. Thus, we distinguished consistently familiar or semantic memories (KK), consistently episodic memories (RR) and those that were initially episodic and became later semantic (RK). Distracter stimuli were different between the two recognition sessions. The pictures used in the figure are not the original IAPS images, but similar items used for illustrative purposes only.

Nevertheless, a review of studies having investigated remote memory in patients with medial temporal lobe amnesia or in animals with hippocampal damage reveals that there are as many reports of ungraded retrograde amnesia as there are with a temporal gradient, thus equally supporting (or contradicting) the standard model of consolidation and the Multiple Trace Theory [7]. However, a crucial dimension to take into account is the episodic or semantic quality of the memories showing or not a temporal gradient in forgetting, and how and to what extent the hippocampus is similarly involved. In order to clarify this issue, we investigated using functional magnetic resonance imaging (fMRI), the temporal evolution of hippocampal and cortical activity underlying the retrieval of recent and remote episodic and semantic memories (i.e., memories retrieved without contextual details and re-experiencing of the learning episode) over a 3-month period. To do so, 18 young healthy participants learned a series of pictures with neutral or emotional valence (Figure 1), and then had to determine during two fMRI sessions taking place 3 days and 3 months after learning whether and how they recognize or not each picture using the “Remember/Know” paradigm [10]. This latter procedure was used to distinguish genuine episodic memories from familiar or semantic ones. Indeed, for some authors, Remember and Know responses reflect the state of consciousness that accompanies retrieval in episodic and semantic memory respectively [11]. Even if other studies suggest that the Remember and Know responses may rather reflect different degrees of recollection [12], this study fits into the theoretical background proposed by Tulving [11].

In the present study, the same learned (“old”) images were presented at both delays allowing us tracking the fate of memories and their possible qualitative change over time. Consequently, we distinguished two types of memories in the fMRI analyses, based on the combination of responses at both delays: those that were consistently episodic at both delays (RR responses) and those that were initially episodic and later familiar or semantic (RK responses). We hypothesized that the hippocampus will remain activated whatever the delay in the case of remaining episodic (RR) memories whereas its contribution will tend to decrease with time for memories becoming semantic (RK). We also hypothesized that RR and RK memories will share common neocortical substrates, notably in frontal areas.

## Materials and Methods

### Subjects

Eighteen right handed volunteers (10 males, 8 females, mean age ± SD: 22.6±1.98 years) gave their written informed consent to take part in this study, which was approved by the Regional Ethics Committee (Comité de Protection des Personnes, Nord-Ouest III). The present study was conducted according to the principles expressed in the Declaration of Helsinki.

No participant had any history of trauma or of medical, psychiatric or sleep disorder, or was on medication. Structural MRI was normal on visual inspection.

### Experimental material

#### Stimuli

Emotional (positive and negative) and neutral pictures from the International Affective Pictorial System (IAPS) database were used [13]. This type of material was chosen since it is well established that emotion reinforces encoding and consolidation processes [14], [15], thus increasing the chance to obtain a satisfactory recognition rate at the 3-month delay to conduct fMRI analyses. Stimuli consisted of 312 pictures (104 positive, valence range on a 9-point scale from the IAPS database: 6.7–8.5; 104 negative, valence range 1.5–3.9 and 104 neutral, valence comprised between 4.2 and 6.5). 192 pictures were presented during the learning phase while the remaining 120 were used as distracters during the recognition sessions.

Positive and negative pictures were equally arousing (p>0.26) and both were more arousing than the neutral pictures (positive, mean arousal: 4.798±0.66; negative, mean arousal: 4.932±0.56; neutral, mean arousal: 3.909±0.78; p<0.001).

Two sets of 96 images were created from the initial set of 192 images and subjects were presented only one of these two sets during the recognition sessions.

#### Experimental Procedure

The experimental design (illustrated in Figure 1) was adapted from Sterpenich et al. [16]. The memory task was developed using the E-Prime software (Psychology Software Tools, 248, Pittsburgh, PA) implemented within IFIS (Invivo, Orlando, FL). Each subject performed a learning session and was scanned using fMRI during two recognition tasks taking place 3 days and 3 months after encoding to assess mid-term and long-term memory consolidation (mean delay ± SD: 85.8±4.7 days).

During the learning phase (day 1), subjects had to memorize intentionally 192 pictures (64 in each valence) displayed on a computer's screen. The pictures were presented only once to participants, one after the other. The task began by the presentation of a white fixation cross on the centre of the screen during 1.5 seconds, followed by the presentation of a picture during 3 seconds. After the picture disappeared, subjects were invited to rate, by pressing on a keypad, its emotional valence (positive, negative or neutral) in no more than 4.5 seconds, and then the next image was displayed. Pictures were presented in a pseudo-random order for each individual.

The first fMRI scanning session took place 3 days after learning (day 4). During this session, subjects were presented 156 emotional or neutral pictures repartitioned in two functional runs (lasting approximately 9 minutes each). Ninety-six pictures (1/3 positive, 1/3 negative and 1/3 neutral) were previously presented during the learning phase (“old” items), whereas 60 other pictures not presented during the learning phase were used as “new” items or distracters. Each picture was displayed for 2 seconds. Participants had then a maximum of 3 seconds to decide, by pressing on a keypad, if they had already seen this picture during the learning phase or not. Four response choices were proposed: “Remember”, “Know”, “Guess” or “New”. A “Remember” response indicated that the recognition of the picture was associated with the retrieval of specific details, feelings or ideas that were present at encoding (i.e., “I remember this picture because when I saw it, it made me think about my trip to Mexico in July 2006”), thus characteristic of an episodic memory recollection. A “Know” response was associated with the mental awareness that the picture has been encoded, but memory retrieval was lacking any further specific details and was based on a mere feeling of familiarity. A “Guess” response referred to the fact that the subject had low confidence in his/her memory for that item. Finally, a “New” response was given when the participant had never seen the picture before.

At the end of each fMRI session, all pictures that received an R response during recognition in the scanner were presented again in a quiet room. Participants were invited to justify their judgment for these pictures with contextual details, perceptions or thoughts. Only R judgments justified by specific contextual details were used in subsequent behavioral and fMRI analyses. An identical procedure was used 3 months later. Note that the same learned (“old”) pictures were used for both retrieval sessions allowing us to track the fate of these memories over time. Distracter stimuli (i.e., “new” items) were different between the two recognition sessions.

### fMRI data acquisition

All images were acquired using a Philips 3T system (Eindhoven, The Netherlands). For each participant, a high resolution T1-weighted anatomical image was acquired first using a 3D fast field echo sequence (3D-T1-FFE sagittal, TR = 20 ms; TE = 4.6 ms; flip angle = 20°; 170 slices; slice thickness = 1 mm; FOV = 256×256 mm^2^; matrix = 256×256; voxel size = 1×1×1 mm^3^), followed by a high resolution T2-weighted anatomical image (2D-T2-SE sagittal, SENSE factor = 2; TR = 5500 ms; TE = 80 ms; flip angle = 90°; 81 slices; slice thickness = 2 mm; FOV = 256×256 mm^2^; matrix = 256×256; voxel size = 2×1×1 mm^3^) and a non-EPI T2* image (2D-T2 Star-FFE axial, SENSE factor = 2; TR = 3505 ms; TE = 30 ms; flip angle = 90°; 70 slices; slice thickness = 2 mm; FOV = 256×256 mm^2^; matrix = 128×128; voxel size = 2×2×2 mm^3^).

Functional images were acquired using an interleaved 2D T2* EPI sequence designed to reduce geometric distortions and magnetic susceptibility artifacts (2D-T2*-FFE-EPI axial, SENSE factor = 2; TR = 2382 ms, TE = 30 ms; flip angle = 80°; 44 slices; slice thickness = 2.8 mm; matrix = 80×80; FOV = 224×224 mm^2^; acquisition voxel size = 2.8×2.8×2.8 mm^3^).

The 420 functional volumes were collected in two runs of 210 volumes each, separated by a short interval (1–2 minutes) during two functional sessions (3 days/3 months).

Stimuli were displayed with a video projector onto a screen installed in front of the scanner. Participants viewed the stimuli through an angled mirror positioned immediately in front of their eyes.

### fMRI data analyses

Functional volumes were pre-processed and analyzed using SPM5 (www.fil.ion.ucl.ac.uk), implemented in Matlab 7.4 (Mathworks Inc., MA). The six initial volumes of each session were discarded to avoid magnetic saturation effects.

During preprocessing steps, EPI images were corrected for slice timing and then realigned on the first volume of the first run. Subsequently, the T1 image was coregistered onto the EPI volumes in 3 steps: (1) coregistration of the non-EPI T2* onto to the mean EPI image of the two runs, (2) coregistration of the T2 image onto the coregistered non-EPI T2* volume, and finally (3) coregistration of the T1 volume onto the coregistered T2 image. The mean EPI image was warped to roughly match the non-EPI T2* volume using the methodology developed and validated by Villain et al. [17] to reduce geometrical distortions. Warping parameters were then applied to all the EPI volumes of the session. The T1 image was then segmented/normalised using the standard SPM5 procedure [18], with ICBM/MNI priors and resulting normalisation parameters applied to the T1 and to the warped images. The normalised EPI-images were finally smoothed at 8 mm FWHM. High-pass filter was implemented using a cut off period of 128 s to remove low frequency drift from time series.

Data were analysed using a mixed-effects model aimed at showing a stereotypical effect in the population from which the subjects were drawn [19]. For each subject, a first-level intra-individual analysis aimed at modelling the data to partition observed neurophysiological responses into components of interest, confounds and error, using a general linear model [20]. As we aimed at tracking the neural substrates of memories over time as a function of the evolution or not of their status from episodic to semantic (or familiar), fMRI data were modelled taking into account the responses provided by the participants at each recognition session. Thus, eight conditions were modelled: R-R (R responses at 3 days that remained R responses at 3 months i.e. consistently episodic); R-K (R responses at 3 days that became K responses at 3 months i.e. initially episodic, later familiar or semantic); R-G (R responses at 3 days that became G responses at 3 months); K-K (K responses at both delays, i.e. consistently familiar or semantic); Correct Rejections (new items that were categorized as new); Misses (old items categorized as new), False Alarms (new items categorized as old (R, K or G) and “other behaviors” (such as K responses at 3 days becoming Guess at 3 months, or any other combination not accounted in the other categories). fMRI analyses were not conducted according to the emotional valence of the pictures. Indeed, distinguishing between negative, positive and neutral trials would have resulted in an insufficient number of events in each condition. Trial length was defined as the time between the onset of the presentation of each picture and the subject's response. The mean number of trials per condition (± SD) was 23.6±17.4 for the RR condition, 12.9±7.2 for the RK condition, 6.1±6.4 for the KK condition and 53.6±4.2 for Correct Rejections. Other response types were rare (KR: 1.17±1.47; KG: 7.39±5.44; RG: 4.4±3.47; GR: 0.22±0.55; GK: 1.5±1.62; GG: 5.67±3.31; Misses: 20.9±12.09; False Alarms: 6.61±4). These data were entered in the individual design matrix to reduce unexplained, residual variance but will not be tested further. For each experimental condition, the regressors of interest were built using stick functions convolved with the canonical hemodynamic response function. Movement parameters estimated during realignment (translations in x, y and *z* directions and rotations around *x-*, *y-* and *z-*axes) were included in the design matrix as variables of no interest. Serial autocorrelations were estimated with a restricted maximum likelihood algorithm, using an autoregressive model of order 1 (+ white noise). Effects of interest for each condition (RR and RK responses) were first tested within each individual by linear contrasts, generating statistical parametric maps [SPM(T)]. These summary statistics images were then entered in a second level, group analysis, corresponding to a random effects (RFX) model, consisting in an analysis of variance (ANOVA) with condition (RR *vs* RK responses) and delay (3 days *vs* 3 months) as within-subject variables. These analyses were performed within the whole group of participants and in a sub-group of participants including those with at least 10 trials per condition (15 and 14 subjects for the RR and RK conditions respectively). As the two analyses provided similar results, we report those obtained on the whole group of participants.

Corrections for multiple testing were applied where mentioned by using either the family-wise error correction over the whole brain (FWE) or over small spherical volumes of interest (SVC; radius 10 mm) around *a priori* locations of anatomical structures of interest derived from the literature and reported in each table.

Region-of-interest (ROI) analyses were also conducted based on *a priori* hypotheses concerning the role of the hippocampus in encoding and retrieval [21] as well as in memory consolidation [8], [9]. Hippocampal ROIs were delineated manually, by a single expert (R.L.J; [22]), on the mean normalized MR image of the whole group of subjects, to ensure that ROIs fit well with our functional data. Delineation was based on both coronal and sagittal slices, according to the procedure described by Pruessner et al. [23]. To account for subtle inter-individual differences in hippocampal size and shape that remained after the spatial normalisation procedure, the ROIs were then applied and adapted to each individual normalized image of grey matter. Then, the mean signal value within hippocampal ROIs was extracted for each subject and each hemisphere. However, as no hemispheric difference was observed, reported mean values are pooling both hemispheres. In addition, to further investigate the topographical distribution of activation within the hippocampus for recent and remote episodic memories (RR responses), hippocampal ROIs were subdivided into the anterior (head) and the posterior (body and tail) parts using Anatomist software. This last delineation was performed on coronal slices only, with the most anterior slice of the posterior hippocampus corresponding to the slice just after the disappearance of the uncus [23], [24].

## Results

### Behavioral Results

3 days after learning, mean recognition performance (correct recognitions associated with R, K or G response) was 84.2±10.1% (mean ± SD). Three months later, performance significantly decreased (70.5±16.7%; t(17) = 5.44, p<0.001) but was still far above chance level (t(17) = 2.27, p<0.05).

A repeated measures ANOVA with response (R *vs* K) and delay (3 days *vs* 3 months) as repeated measures factors was conducted on memory performance. This analysis revealed a significant effect of the response type (F(1,17) = 6.82, p<0.02). Thus, participants provided more R responses than K ones, whatever the delay. The effect of delay was also significant (F(1,17) = 38.97, p<0.001) resulting, as expected, in better memory performance during the first recognition session (3 days) than during the second one (3 months). The response by delay interaction was also significant (F(1,17) = 17.75, p<0.001). Tukey's *post hoc* comparisons revealed that participants provided more R responses at 3 days (mean ± SD: 44±16.7%) than after 3 months (27.9±18.8%; p<0.001). Conversely, the proportion of K responses remained unchanged over time (3 days: 21.5±11.5%; 3 months: 21.6±12.2%; all p values >0.12). Finally, participants gave significantly more R than K responses 3 days after learning (p<0.001), but this difference was no longer significant after 3 months (p>0.99).

Then, one-way ANOVAs were conducted on the discrimination score (d′), reflecting recognition accuracy, and the response criterion (C). The discrimination score d′ was calculated on correct recognitions associated with a R, K or Guess response. These analyses revealed that the discrimination score did not change over time (mean d′ ± SD: 3 days: 2.35±0.52; 3 months: 2.19±0.54; F(1,17) = 1.64, p>0.21) whereas the response criterion increased with delay (mean C ± SD 3 days: 0.02±0.43; 3 months: 0.49±0.43; F(1,17) = 26.3, p<0.001), indicating that participants became more conservative in their judgments over time.

When taking into account the evolution of memory traces with time and distinguishing consistently episodic memories (RR responses), from initially episodic, later familiar or semantic (RK responses) and consistently semantic memories (KK responses), mean subjects' performance, calculated on the total number of old items (n = 96), was 24.5±18.2% for the RR condition, 13.5±7.5% for the RK condition and 6.3±6.7% for the KK condition. 80% of RR memories corresponded to emotionally-laden pictures (with as many positive as negative pictures). Almost 80% of RK memories and nearly 60% of KK memories have an emotional valence, equally distributed between positive and negative items in both cases.

As we were interested in the comparison between RR and RK responses, we compared the percentage of emotionally-laden pictures for each response type using a repeated-measures ANOVA. This analysis revealed a significant effect of emotion (F(2,34) = 7.22, p<0.01), but no effect of the response type (F(1,17) = 0.11, p>0.74). The interaction between the two factors was not significant (F(2,34) = 0.08, p>0.92), ruling out any confounding effect of emotional valence.

### Brain Imaging Data

#### Neural networks activated during the retrieval of RR and RK memories

**Figure 2:**
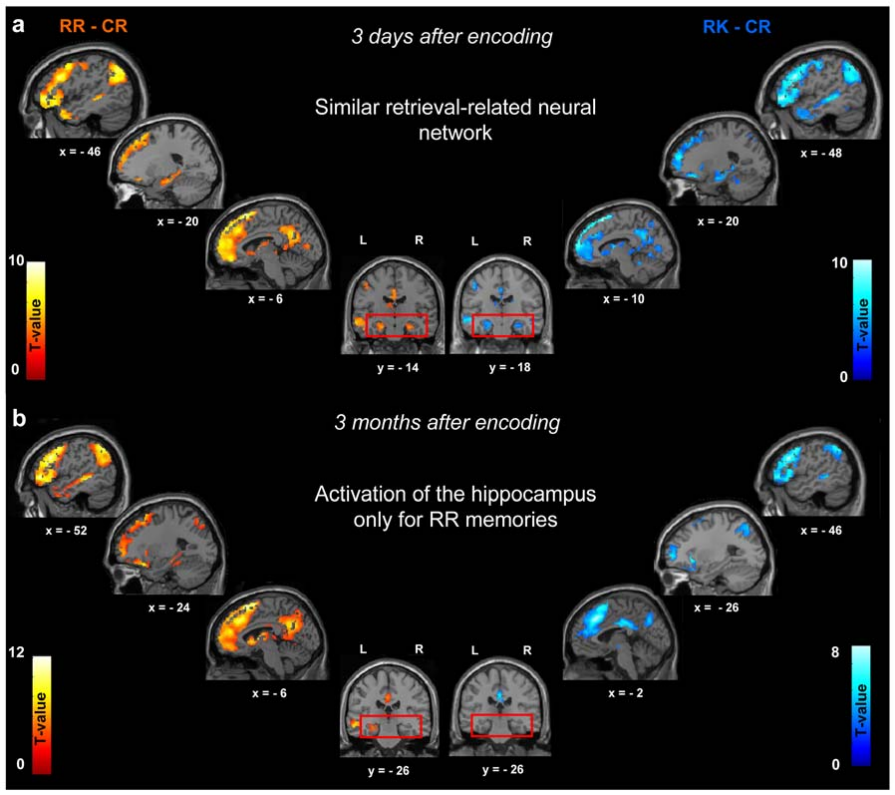
Brains areas subserving retrieval of consistently episodic (RR responses) or initially episodic and later semantic (RK responses) memories compared to correct rejections (CR), 3 days (a) and 3 months (b) after encoding. Activations are displayed at p<0.001 (uncorrected) on sagittal or coronal sections of the MNI template. Note that the right hippocampal activation did not survive correction for multiple comparisons.

Whole-brain analyses revealed that, compared to correct rejections of new items, RR memories activated, at both delays, a large neural network including frontal and lateral temporal cortices, the caudate nucleus, the thalamus, the hippocampus and retrorolandic areas (precuneus, inferior parietal lobule and posterior cingulate cortex; Figure 2; Tables 1 and 2, p<0.05, corrected for multiple comparisons). No higher activity was found after 3 days than after 3 months. In contrast, higher BOLD responses after 3 months than after 3 days were observed in the superior frontal gyrus and the dorsolateral prefrontal cortex (p^svc(10 mm)^<0.05; Table 3). On day 4, RK (memories that were initially episodic and then became semantic) and RR memories showed a similar pattern of activation (Figure 2; Tables 1 and 4). After 3 months, significant activity for RK memories was observed mainly in frontal and posterior parietal areas (p<0.05, corrected for multiple comparisons; Figure 2; Table 5), but no hippocampal activation was detected. Additionally, higher BOLD responses were found after 3 days than after 3 months in the hippocampus, the amygdala and in the precentral and fusiform gyri (Table 6; p<0.05, corrected for multiple comparisons). No suprathreshold cluster was reported in the reverse contrast (Table 6). As reported in the Methods section, the mean number of trials was inferior in the RK condition (mean ± SD: 12.9±7.2) than in the RR condition (23.6±17.4). In this respect, the lack of hippocampal activation at the 3-month delay for RK responses could be due to a lack of power in statistical analyses. To answer this issue, another analysis was conducted matching, for each participant, the number of trials in RR and RK conditions. RR trials that were not included in the analysis were randomly selected in the course of the functional runs. This analysis revealed broadly similar results as reported above with the total number of trials (see Tables S1, S2, S3, S4 and S5) and confirmed that hippocampal activity decreases over time when memories become semantic or familiar.

**Table 1:** Brain areas associated to the retrieval of consistently episodic memories (RR responses) compared to correct rejections at the 3-day delay.

Side	Anatomical region	cluster size	x	y	z	Z	p value	*Reference*
L	Inferior frontal gyrus	1737	−48	40	−4	7.77	<0.001*	
L	Middle frontal gyrus		−44	6	46	6.68	<0.001*	
L	Superior frontal gyrus	1945	−6	20	64	7.3	<0.001*	
L	Medial frontal gyrus		−4	48	−12	6.23	<0.001*	
L	Middle temporal gyrus	574	−60	−34	−10	7.02	<0.001*	
L	Inferior parietal lobule	1097	−46	−62	42	6.82	<0.001*	
L	Angular gyrus		−50	−62	26	6.52	<0.001*	
L	Superior temporal gyrus	121	−40	20	−30	6.26	<0.001*	
R	Medial orbital frontal gyrus	251	2	48	−12	5.79	<0.001*	
L	Precuneus	268	−4	−56	30	5.7	<0.001*	
L	Posterior cingulate gyrus		−10	−50	34	5.66	<0.001*	
L	Ventromedial prefrontal cortex	233	−4	42	−10	5.26	<0.001**	[*39*]
R	Anterior cingulate gyrus	12	6	14	26	5.03	0.009*	
L	Inferior temporal gyrus	10	−54	−2	−32	5.02	0.009*	
L	Caudate nucleus	17	−12	12	12	4.9	0.016*	
L	Hippocampus	215	−22	−10	−20	4.45	0.001**	[*39*]
L	Thalamus	56	−6	−10	6	3.86	0.007**	[*25*]

x, y, z = coordinates in mm in the Montreal Neurological Institute space. All regions listed are statistically significant at p<0.05 (FWE corrected, *) or p_svc_<0.05 (**), after correction in a small spherical volume (10 mm) around coordinates previously reported in the literature (specified in the last column). For brevity, each region is listed only once; when several peaks were observed in the same region, the coordinates refer to the strongest activation. Minimum cluster size: 10 contiguous voxels.

**Table 2:** Brain areas associated to the retrieval of consistently episodic memories (RR responses) compared to correct rejections at the 3-month delay.

Side	Anatomical region	cluster size	x	y	z	Z	p value	*Reference*
L	Inferior frontal gyrus	7025	−52	20	32	Inf	<0.001*	
L	Inferior parietal lobule	1554	−38	−60	48	Inf	<0.001*	
R	Caudate nucleus	877	12	8	14	7.41	<0.001*	
L	Caudate nucleus		−8	2	12	6.86	<0.001*	
L	Middle temporal gyrus	480	−56	−38	−6	7.22	<0.001*	
L	Precuneus	806	−10	−50	34	6.55	<0.001*	
L	Posterior cingulate gyrus		−6	−54	28	6.52	<0.001*	
R	Supplementary motor area	172	4	14	62	6.4	<0.001*	
R	Medial frontal gyrus		10	20	62	6.06	<0.001*	
R	Superior frontal gyrus		18	18	64	4.81	0.024*	
L	Superior temporal gyrus	56	−44	14	−32	6.33	<0.001*	
L	Thalamus	206	−6	−10	6	6	<0.001**	[*25*]
R	Cerebellum	26	30	−62	−28	5.36	0.002*	
L	Ventromedial prefrontal cortex	233	−4	42	−10	5.26	<0.001**	[59]
R	Retrosplenial cortex	31	4	−50	16	4.67	0.043*	
L	Hippocampus	226	−26	−24	−14	4.48	0.001**	[*59*]

x, y, z = coordinates in mm in the Montreal Neurological Institute space. All regions listed are statistically significant at p<0.05 (FWE corrected, *) or psvc<0.05 (**), after correction in a small spherical volume (10 mm) around coordinates previously reported in the literature (specified in the last column). For brevity, each region is listed only once; when several peaks were observed in the same region, the coordinates refer to the strongest activation. Minimum cluster size: 10 contiguous voxels.

**Table 3:** Brain areas in which activity during the retrieval of consistently episodic memories (RR) compared to correct rejections is higher after the 3-day than the 3-month delay or conversely.

Side	Anatomical region	cluster size	x	y	z	Z	p_svc_	*Reference*
***3 days>3 months***								
			*No suprathreshold clusters*					
***3 months>3 days***								
L	Superior frontal gyrus	128	−20	14	64	3.49	0.023	[*30*]
L	Dorsolateral prefrontal cortex	312	−44	36	26	3.4	0.029	[*25*]

x, y, z = coordinates in mm in the Montreal Neurological Institute space. All regions listed are statistically significant at p_svc_<0.05, after correction in a small spherical volume (10 mm) around coordinates previously reported in the literature (specified in the last column).

**Table 4:** Brain areas associated to the retrieval of initially episodic, later semantic memories (RK responses) compared to correct rejections at the 3-day delay.

Side	Anatomical region	cluster size	x	y	z	Z	p value	*Reference*
L	Inferior frontal gyrus	3843	−48	40	−4	7.81	<0.001*	
L	Superior frontal gyrus		−6	20	62	7.42	<0.001*	
L	Inferior parietal lobule	1081	−34	−74	38	6.79	<0.001*	
L	Middle temporal gyrus	630	−60	−34	−10	6.4	<0.001*	
L	Temporal pole	54	−44	12	−30	6.06	<0.001*	
L	Middle frontal gyrus	299	−30	54	12	5.69	<0.001**	[*25*]
L	Precuneus	229	−4	−56	30	5.59	0.001*	
L	Posterior cingulate gyrus		−2	−50	18	5.44	0.001*	
R	Medial frontal gyrus	100	2	62	10	5.55	0.001*	
L	Insula	38	−30	14	0	5.53	0.001*	
R	Caudate nucleus	74	14	10	12	5.43	0.001*	
L	Caudate nucleus	10	−8	0	10	5.34	0.002*	
L	Precentral gyrus	35	−48	−14	48	5.3	0.002*	
L	Medial orbital frontal gyrus	46	−2	50	−12	5.28	0.003*	
R	Anterior cingulate gyrus	21	6	28	20	5.17	0.004*	
L	Middle cingulate gyrus	29	−2	−10	34	5.16	0.005*	
R	Cerebellum	26	32	−68	−26	5.1	0.006*	
R	Temporal pole	24	46	16	−34	5.09	0.007*	
R	Medial orbital frontal gyrus	31	2	48	−14	5.04	0.008*	
L	Hippocampus	10	−22	−12	−22	4.83	0.021*	
L	Ventromedial prefrontal cortex	161	−2	42	−10	4.56	<0.001*	[*39*]
L	Amygdala	301	−22	0	−28	3.99	0.030**	[*25*]
R	Fusiform gyrus	225	44	−68	−18	3.34	0.035**	[*33*]

x,y,z = coordinates in mm in the Montreal Neurological Institute space. All regions listed are statistically significant at p<0.05 (FWE corrected, *) or psvc<0.05 (**), after correction in a small spherical volume (10 mm) around coordinates previously reported in the literature (specified in the last column). For brevity, each region is listed only once; when several peaks were observed in the same region, the coordinates refer to the strongest activation. Minimum cluster size: 10 contiguous voxels.

**Table 5:** Brain areas associated to the retrieval of initially episodic, later semantic memories (RK responses) compared to correct rejections at the 3-month delay.

Side	Anatomical region	cluster size	x	y	z	Z	p value	*Reference*
L	Inferior frontal gyrus	1684	−52	20	32	7.61	<0.001*	
L	Middle frontal gyrus		−44	6	44	6.68	<0.001*	
L	Medial frontal gyrus	644	−4	30	40	7.32	<0.001*	
L	Supplementary motor area		−4	22	58	6.5	<0.001*	
L	Superior frontal gyrus		−16	16	62	6.22	<0.001*	
R	Medial frontal gyrus	57	2	30	40	6.49	<0.001*	
R	Superior frontal gyrus	45	18	18	64	5.93	<0.001*	
L	Inferior parietal lobule	571	−46	−54	50	5.64	<0.001*	
R	Caudate nucleus	125	12	12	10	6.01	<0.001*	
	Posterior cingulate gyrus	171	0	−24	36	5.68	<0.001**	[60]
L	Middle temporal gyrus	63	−58	−40	−4	5.33	0.002*	
	Middle cingulate gyrus	106	0	−20	38	5.27	0.003*	
L	Middle cingulate gyrus		−2	−18	30	5.02	0.009*	
R	Anterior cingulate gyrus	19	6	38	16	5.18	0.004*	
L	Precuneus	22	−8	−64	32	5.13	0.005*	
L	Caudate nucleus	38	−8	10	0	5.11	0.006*	
L	Angular gyrus	106	−44	−60	36	5.02	<0.001**	[61]
R	Superior parietal lobule	266	34	−68	48	4.61	0.002**	[*61*]
	Retrosplenial cortex	122	0	−38	24	3.87	0.007**	[62]

x, y, z = coordinates in mm in the Montreal Neurological Institute space. All regions listed are statistically significant at p<0.05 (FWE corrected, *) or psvc<0.05 (**), after correction in a small spherical volume (10 mm) around coordinates previously reported in the literature (specified in the last column). For brevity, each region is listed only once; when several peaks were observed in the same region, the coordinates refer to the strongest activation. Minimum cluster size: 10 contiguous voxels.

**Table 6:** Brain areas in which activity during the retrieval of initially episodic, later semantic memories (RK) compared to correct rejections is higher after the 3-day than the 3-month delay or conversely.

Side	Anatomical region	cluster size	x	y	z	Z	p value	*Reference*
***3 days>3 months***								
L	Hippocampus	64	−22	−10	−20	5.9	<0.001*	
R	Amygdala	12	24	−8	−18	4.91	0.015*	
R	Precentral gyrus	199	50	−14	46	4.74	0.001**	[63]
R	Fusiform gyrus	343	42	−58	−12	4.09	0.007**	[*63*]
***3 months>3 days***								
			*No suprathreshold clusters*					

x, y, z = coordinates in mm in the Montreal Neurological Institute space. All regions listed are statistically significant at p<0.05 (FWE corrected, *) or psvc<0.05 (**), after correction in a small spherical volume (10 mm) around coordinates previously reported in the literature (specified in the last column). For brevity, each region is listed only once; when several peaks were observed in the same region, the coordinates refer to the strongest activation. Minimum cluster size: 10 contiguous voxels.

**Figure 3:**
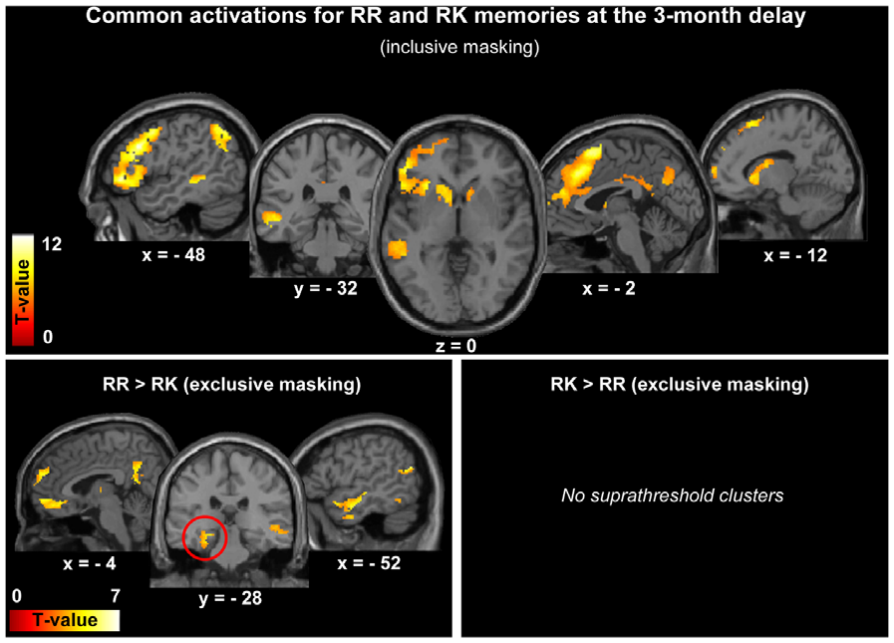
Common and specific activations for RR and RK memories, 3 months after encoding. Common activations (top) are displayed at p<0.05 (FWE corrected) and specific activations (bottom) at p<0.001 (uncorrected), on axial, sagittal and coronal slices of the MNI template. The red circle illustrates the specific activation of the hippocampus for RR memories.

To determine whether RR and RK responses (compared to correct rejections) recruited the same neocortical areas after 3 months, the contrast [RR-CR] was masked by the contrast [RK-CR] (inclusive masking at p<0.001). This analysis revealed a common network of activation in frontal and posterior parietal cortices, in the middle temporal gyrus as well as in the caudate nucleus and thalamus (p<0.05, FWE corrected; Figure 3; Table 7). Besides these commonalities, significant BOLD responses were observed for RR memories, but not for RK ones (exclusive masking at p<0.05), in the superior frontal gyrus and the ventromedial prefrontal cortex, the inferior parietal lobule and precuneus, the lateral temporal cortex and the hippocampus (p<0.05, corrected for multiple comparisons, Figure 3; Table 8). No specific activation was observed for RK memories compared to RR ones.

**Table 7:** Brain areas commonly activated at 3 months for to the retrieval of consistently episodic memories (RR) and initially episodic, then semantic memories (RK) (inclusive masking at p<0.001).

Side	Anatomical region	cluster size	x	y	z	Z	p _corr (FWE)_
L	Inferior frontal gyrus	5142	−52	20	32	Inf	<0.001
L	Superior frontal gyrus		−6	20	62	Inf	<0.001
L	Inferior parietal lobule	1048	−38	−60	48	Inf	<0.001
R	Caudate nucleus	326	12	8	14	7.41	<0.001
L	Middle temporal gyrus	311	−56	−38	−6	7.22	<0.001
R	Medial frontal gyrus	412	2	30	40	7.1	<0.001
R	Anterior cingulate gyrus		8	38	18	5.93	<0.001
L	Caudate nucleus	421	−8	2	12	6.86	<0.001
L	Thalamus		−8	−10	8	6.5	<0.001
L	Posterior cingulate gyrus	370	−10	−50	34	6.55	<0.001
L	Precuneus		−6	−54	28	6.52	<0.001
R	Supplementary motor area	149	4	14	62	6.4	<0.001
R	Superior frontal gyrus		18	18	64	4.81	<0.001

x, y, z = coordinates in mm in the Montreal Neurological Institute space. For brevity, each region is listed only once; when several peaks were observed in the same region, the coordinates refer to the strongest activation. Minimum cluster size: 10 contiguous voxels.

**Table 8:** Brain areas in which activity at 3 months is specifically activated during the retrieval of consistently episodic memories (RR) but not for initially episodic, then semantic memories (RK) and conversely.

Side	Anatomical region	cluster size	x	y	z	Z	p value	*Reference*
***RR>RK (exclusive masking at p<0.05)***								
L	Superior frontal gyrus	50	−8	54	20	6.09	<0.001*	
L	Ventromedial prefrontal cortex	112	−2	52	−8	5.63	<0.001*	
L	Inferior parietal lobule	22	−44	−58	22	5.62	<0.001*	
L	Middle temporal gyrus	19	−54	−66	22	5.36	0.002*	
R	Middle temporal gyrus	10	48	−34	−2	5.04	0.008*	
R	Ventromedial prefrontal cortex	17	2	52	−6	5	0.013*	
L	Temporal pole	15	−36	20	−28	5.51	0.001*	
L	Precuneus	11	−2	−58	22	5.05	0.008*	
L	Hippocampus	226	−26	−24	−14	4.48	0.001**	[*59*]
***RK>RR (exclusive masking at p<0.05)***								
			*No suprathreshold clusters*					

x, y, z = coordinates in mm in the Montreal Neurological Institute space. All regions listed are statistically significant at p<0.05 (FWE corrected, *) or psvc<0.05 (**), after correction in a small spherical volume (10 mm) around coordinates previously reported in the literature (specified in the last column). For brevity, each region is listed only once; when several peaks were observed in the same region, the coordinates refer to the strongest activation. Minimum cluster size: 10 contiguous voxels.

#### ROI analyses

**Figure 4:**
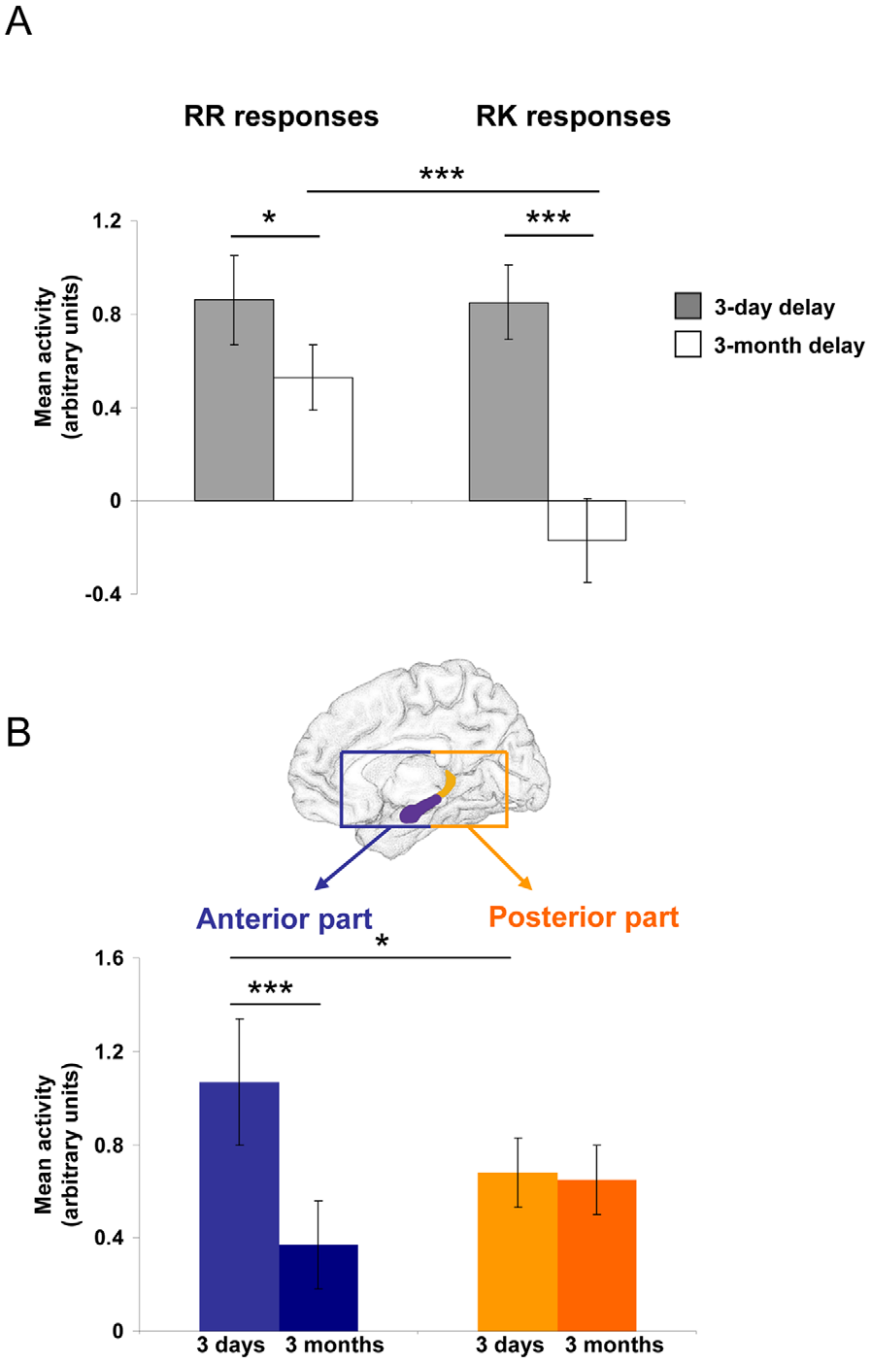
Results of ROI analyses in the hippocampus. A. Hippocampal activity (mean ± SEM) according to the nature of memories (RR *vs* RK responses) and delay (3 days *v*s 3 months). *: p<0.05, ***: p<0.001. B. Hippocampal activity (mean ± SEM) for RR responses according to the delay (3 days *vs* 3 months) distinguishing the anterior and posterior parts. ***: p<0.001.

A first ROI analysis focusing on the whole hippocampus was conducted using an ANOVA with response (RR *vs* RK) and delay (3 days *vs* 3 months) as within-subjects factors. This analysis revealed a significant effect of delay (F(1,17) = 9.46, p<0.01) and response (F(1,17) = 7.84, p<0.05) and a significant interaction between both factors (F(1,17) = 18.73, p<0.001). Tukey's *post hoc* comparisons revealed that activity in this area significantly decreased over the 3-month interval for RR responses (3 days (mean ± SEM): 0.86±0.19; 3 months 0.53±0.16, p<0.05; Figure 4A). Hippocampal activity at 3 days was not different for RK (0.85±0.14) and RR responses (p>0.99) but was significantly larger for RR responses than for RK ones (−0.17±0.18) after 3 months (p<0.001; Figure 4A). Additionally, hippocampal activity was significantly reduced after a 3-month delay for RK responses (p<0.001; Figure 4A).

Finally, based on the hypothesis that the anterior and posterior parts of the hippocampus may subserve different memory functions [21], [25], we conducted a supplementary analysis dividing the hippocampus in two parts along the rostrocaudal axis. This analysis did not reveal any significant effect of delay (F(1,17) = 1.87, p>0.19) nor of the part of the hippocampus (F(1,17) = 0.35, p>0.56), but a significant interaction between both factors (F(1,17) = 12.01, p<0.01). Tukey's *post hoc* comparisons revealed that the anterior hippocampus was more activated for recent than for remote memories (3 days: 1.07±0.27 (mean ± SEM); 3 months: 0.37±0.19; p<0.001; Figure 4B). In contrast, the activity in the posterior part was equivalent for recent and remote episodic memories (3 days: 0.68±0.15; 3 months: 0.65±0.15; p>0.99; Figure 4B). Finally, the anterior hippocampus was more activated after 3 days than the posterior part (p<0.05). This difference was not present any more after 3 months (p>0.21).

## Discussion

Understanding how recent and remote memories are reorganized and stored within neural networks is an exciting challenge for neuroscientists. This question was already addressed by Viskontas et al. [26] who investigated the neural substrates of memory retrieval for items learned one week before, but the authors restricted their analyses to the medial temporal lobe. Our prospective fMRI study is therefore the first to investigate, on the whole brain, the reorganization of episodic memories and memories that became semantic over a relatively long retention interval of 3 months. Our data show that hippocampal activation remains stable over time, at least in the posterior part of this region, for episodic memories whereas it decreases for those that became semantic over time and were retrieved without the recollection of specific contextual details.

We will first discuss the role of the hippocampus in the retrieval of recent/remote episodic and familiar, semantic memories. Then, we will turn toward the pattern of neocortical activations, 3 days and 3 months after learning, highlighting commonalities and specificities between consistently episodic and initially episodic, later semantic memories.

### Role of the hippocampus in the retrieval of recent/remote episodic and semantic memories

Our data show that the hippocampus maintains activity over time for the retrieval of episodic memories only. This finding is consistent with studies conducted in brain-damaged patients, exhibiting non-graded retrograde amnesia for personal memories (e.g., [27], [28]). It is also corroborated by several neuroimaging studies of autobiographical memory in young adults [29], [30]. For instance, Söderlund and collaborators [31] recently showed similar hippocampal activation for the retrieval of personal events across various periods of life extending from the very recent to the remote past (10 years ago). Therefore, as long as they retain their contextual details, retrieval of episodic memories requires the hippocampus. This structure may serve as an “index”, binding together items and their context [32].

Nevertheless, one may argue that hippocampal activation in our study merely reflects re-encoding processes during both recognition sessions. However, the fact that activation of the anterior part of the hippocampus, classically related to encoding processes [21], decreased over time argues against this view. The pattern activation along the rostrocaudal axis of the hippocampus is consistent with previous studies of episodic [33] and autobiographical memory [34]. According to Gilboa et al. [35], this gradient of activation may be related to the richness of re-experiencing (vividness) rather than the age of the memory *per se*.

Surprisingly, retrieval of recent and remote episodic memories elicited activation only in the left hippocampus. Right hippocampal activation was observed, but at a more lenient statistical threshold ([36 −14 −24], z = 3.09, p<0.005 uncorrected for multiple comparisons). In line with our results, Maguire and Frith [36] found that the activation of right and left hippocampi was modulated by the remoteness of autobiographical memories. Thus, the right hippocampus showed a temporal gradient, with activity decreasing for the retrieval of remote memories while the left hippocampus was invariably involved in the retrieval of autobiographical memories across the lifespan. Other data from our laboratory confirmed the involvement of the left hippocampus during retrieval whatever the remoteness of memories, but also indicate that an additional activation of the right hippocampus may characterize rich episodic, autobiographical memory recollection [37]. In our study, we can surmise that, given the high number of pictures to memorize, subjects did not create a representation with specific and rich contextual details for each item during encoding, explaining the lack of right hippocampal activation at a corrected statistical threshold.

In contrast to consistently episodic memories, our data also revealed that hippocampal activation decreased when memories became familiar or semantic over time. This result was not due to a lack of statistical power due to the fewer number of RK trials compared to RR ones as an analysis in which we matched the number of events in each condition revealed the same results (see Tables S1, S2, S3, S4, and S5). This suggests that the simple passage of time modifies the format of memory representations, by reducing the richness of details present during the initial encoding of the event and only retaining the more schematic or semantic aspects of the past. This idea termed the “transformation hypothesis” [38] assumes that the initial, context-specific hippocampal memory is converted to a non-contextual schematic memory, represented in extra-hippocampal areas. Whereas the former type of memory is lost after hippocampal damage, the latter is preserved, thereby accounting for the temporal gradients observed in patients with retrograde amnesia [7]. On a neurobiological point of view, the transformation hypothesis would predict a shift from hippocampal to extra-hippocampal regions as memories loose their contextual features and become familiar or semantic. Thus, this kind of memories may consolidate over time through strengthening of cortico-cortical connections and progressive disengagement of the hippocampal formation. With time, retrieval of these memories may rely only on neocortical areas. However, this result can not be interpreted in light of the standard model of memory consolidation [8] as this model does not mention any qualitative change during the consolidation process. Indeed, according to this model, consolidation entails a process of duplication in which a particular cortically based memory is identical to the hippocampus-dependent memory from which it derived [7].

#### Neocortical activity 3 days after encoding for RR and RK memories

As early as 3 days after learning, retrieval of RR and RK memories activated a large set of neocortical areas including the ventromedial prefrontal and anterior cingulate cortices, as well as posterior parietal areas. At the system level, memory consolidation refers to a gradual and usually slow (lasting up to decades) process of reorganization of the brain regions that support memory [6]. For declarative memories (grouping together episodic and semantic memories), this process implies a dialogue between the hippocampal formation and various neocortical areas. In a prospective fMRI study, Takashima et al. [39] observed a gradual recruitment of the ventromedial prefrontal cortex over one month for the retrieval of remote memories, paralleled by a decrease in hippocampal activity. In our study, we observed a similar activation of the ventromedial prefrontal cortex 3 days after encoding, in line with previous studies that assessed the role of sleep in memory consolidation and probed memory after the same interval [16], [40]. These results, together with other recent studies in animals [41], [42] and in humans [43] challenged the existence of two complementary learning systems with the hippocampus and the neocortex as a fast and a slow learner respectively [44], showing that fast learning can also occur at the cortical level. Indeed, Tse et al. [42] showed that hippocampal-dependent leaning of new paired associates is associated with a striking up-regulation of immediate early genes in prefrontal areas. In the same way, Shtyrov et al. [43] revealed, using event-related potentials, the existence of a cortical correlate of learning emerging within minutes of exposure to new pseudo-words. These data indicate that the dynamics of memory reorganization may be much faster than initially thought.

Given its multiple anatomical connections with limbic areas, entorhinal and perirhinal cortices or with the striatum, the anterior cingulate cortex appears ideally suited to integrate information from distributed areas and would play a role in remote memory close to that initially supported by the hippocampus [45]. However, if prefrontal activations (including the anterior cingulate cortex) were observed during memory retrieval after a 3-day retention interval, we can not exclude that these activations already existed from the outset of the learning process and do not necessarily reflect a transfer towards long term sites of storage. In this alternative view, the activation of prefrontal areas, generally engaged in ‘high order” cognitive functions, could reflect abilities to allocate attentional resources at retrieval or to specify retrieval cues [46], [47], processes supporting on-line monitoring and updating of retrieved information [48], [49], or more strategic aspects of retrieval rather than retrieval *per se* (“working-with memory” hypothesis, [50]).

Activations were also observed in the posterior parietal cortex. This large region, encompassing the precuneus, retrosplenial and posterior cingulate cortices as well as the superior and inferior parietal lobule, is almost consistently reported in studies of episodic memory retrieval [51]. According to a recent meta-analysis, the inferior lateral parietal lobule is associated with retrieval success and subserves the attentional requirements of the tasks [25]. In the same vein, Cabeza and coworkers [52], [53] proposed that the inferior parietal lobule mediates the automatic, bottom-up attentional capture by retrieved memory contents. Posterior parietal areas are also involved in mental imagery, a cognitive process critically engaged during memory retrieval [54].

#### Neocortical activity 3 months after encoding for RR and RK memories

Three months after encoding, retrieval of remote consistently episodic (RR) memories and of initially episodic, later semantic (RK) memories recruited a similar neocortical network, albeit less extended for RK memories. This network encompassed notably the precuneus and inferior parietal lobule, the middle temporal and inferior frontal cortices, as well as the supplementary motor area, the anterior cingulate cortex and/or ventromedial prefrontal cortex. These results are in line with studies that investigated the neural substrates of Remember and Know responses and revealed partially over-lapping neural networks [55]. In addition, some of the brain areas commonly activated in our study were previously identified as elements of a common functional brain network for autobiographical, episodic and semantic memory retrieval [56]. In particular, frontal nodes in this network may subserve top-down attentional processes, inhibitory and monitoring functions, as well as working memory demands that are necessary to achieve memory retrieval. Lateral temporal activity may allow access to general semantic information, a preliminary step essential in the retrieval of all declarative memory, whether they are episodic or semantic [56].

Interestingly, retrieval of remote RR memories, compared to recent ones, elicited higher responses in the superior frontal gyrus and the dorsolateral prefrontal cortex, indicating that the contribution of these two areas to retrieval increases with the remoteness of memories. In a study of autobiographical memory, Piolino et al. [34] reported activations in the dorsolateral prefrontal cortex when comparing recent to remote memories. Albeit apparently discrepant, these results fit however well with our data as the recent period corresponded in this study to memories experienced during the preceding year, and so encompassed the period tested in our study. In addition, Mangels et al. [57] reported that patients with lesions of the dorsolateral prefrontal cortex exhibit impaired remote memory for public information, while recognition was relatively preserved. This suggests that the dorsolateral prefrontal cortex may be involved in strategic search processes of memory which may be more important and/or effortful as memories are remote.

When comparing the pattern of brain activity for retrieval of remote RR and RK memories, significant activations were observed in the prefrontal cortex, the inferior parietal lobule, the lateral temporal cortex, the precuneus and the hippocampus for RR memories, but not for RK ones. The reverse contrast failed to reveal significant clusters. No hippocampal activation was observed for the retrieval of remote RK memories. Thus, retrieval of initially episodic, later semantic memories can be achieved only thanks to neocortical areas. The ventromedial prefrontal cortex, including the anterior cingulate cortex, would be able to hold the role initially played by the hippocampus during memory retrieval, as suggested by previous studies [39], [58]. The disengagement of the hippocampal formation over time is associated with a loss of specific, contextual details. In this respect, this change could reflect a process of semanticization of memories. Nevertheless, as factors such as vividness, amount of details recollected, rehearsal and re-encoding were not controlled and could have influenced our results, further studies are needed to explore more precisely semanticization.

## Conclusions

In conclusion, our data extend earlier findings which were based on short-retrieval intervals and indicate that, in the course of memory consolidation, the gradual strengthening of cortico-cortical connections occurs without dampening the contribution of the hippocampus, at least for episodic memories, providing strong support in favour of the Multiple Trace Theory. At variance, memories becoming familiar or semantic over time consolidate through strengthening of cortico-cortical connections and progressive disengagement of the hippocampus.

## Supporting Information

Table S1 **Brain areas associated to the retrieval of consistently episodic memories (RR responses) and initially episodic, then semantic memories (RK responses) compared to correct rejections at the 3-day delay.** X, y, z refer to coordinates (in mm) in the Montreal Neurological Institute space. All regions listed are statistically significant at p<0.05 (FWE corrected, *) or psvc<0.05 (**), after correction in a small spherical volume (10 mm) around coordinates previously reported in the literature (specified in the last column). For brevity, each region is listed only once; when several peaks were observed in the same region, the coordinates refer to the strongest activation. Minimum cluster size: 10 contiguous voxels.
(DOC)

Table S2 **Brain areas associated to the retrieval of initially episodic, later semantic memories (RK responses) compared to correct rejections at the 3-day delay.** X, y, z refer to coordinates (in mm) in the Montreal Neurological Institute space. All regions listed are statistically significant at p<0.05 (FWE corrected, *) or psvc<0.05 (**), after correction in a small spherical volume (10 mm) around coordinates previously reported in the literature (specified in the last column). For brevity, each region is listed only once; when several peaks were observed in the same region, the coordinates refer to the strongest activation. Minimum cluster size: 10 contiguous voxels.
(DOC)

Table S3 **Brain areas associated to the retrieval of consistently episodic memories (RR responses) compared to correct rejections at the 3-month delay.** X, y, z refer to coordinates (in mm) in the Montreal Neurological Institute space. All regions listed are statistically significant at p<0.05 (FWE corrected, *) or psvc<0.05 (**), after correction in a small spherical volume (10 mm) around coordinates previously reported in the literature (specified in the last column). For brevity, each region is listed only once; when several peaks were observed in the same region, the coordinates refer to the strongest activation. Minimum cluster size: 10 contiguous voxels.
(DOC)

Table S4 **Brain areas associated to the retrieval of initially episodic, then semantic memories (RK responses) compared to correct rejections at the 3-month delay.** X, y, z refer to coordinates (in mm) in the Montreal Neurological Institute space. All regions listed are statistically significant at p<0.05 (FWE corrected, *) or psvc<0.05 (**), after correction in a small spherical volume (10 mm) around coordinates previously reported in the literature (specified in the last column). For brevity, each region is listed only once; when several peaks were observed in the same region, the coordinates refer to the strongest activation. Minimum cluster size: 10 contiguous voxels.
(DOC)

Table S5 **Brain areas in which activity during the retrieval of initially episodic, then semantic memories (RK responses) compared to correct rejections is higher after 3 days than 3 months, and conversely.** X, y, z refer to coordinates (in mm) in the Montreal Neurological Institute space. All regions listed are statistically significant at p<0.05 (FWE corrected, *) or psvc<0.05 (**), after correction in a small spherical volume (10 mm) around coordinates previously reported in the literature (specified in the last column). For brevity, each region is listed only once; when several peaks were observed in the same region, the coordinates refer to the strongest activation. Minimum cluster size: 10 contiguous voxels.
(DOC)
