# Supplementary material for: The Hippocampus Remains Activated over the Long Term for the Retrieval of Truly Episodic Memories
Source: PLoS One. 2012 Aug 24;7(8):e43495. doi: 10.1371/journal.pone.0043495 (PMC3427359; doi:10.1371/journal.pone.0043495)
Supplement: Table S1 — Brain areas associated to the retrieval of consistently episodic memories (RR responses) and initially episodic, then semantic memories (RK responses) compared to correct rejections at the 3-day delay. X, y, z refer to coordinates (in mm) in the Montreal Neurological Institute space. All regions listed are statistically significant at p<0.05 (FWE corrected, *) or psvc<0.05 (**), after correction in a small spherical volume (10 mm) around coordinates previously reported in the literature (specified in the last column). For brevity, each region is listed only once; when several peaks were observed in the same region, the coordinates refer to the strongest activation. Minimum cluster size: 10 contiguous voxels. (DOC) [file pone.0043495.s001.doc]

| **Table S1: Brain areas associated to the retrieval of consistently episodic memories (RR responses) and initially episodic, then semantic memories (RK responses) compared to correct rejections at the 3-day delay.** | | | | | | | | |
| --- | --- | --- | --- | --- | --- | --- | --- | --- |
|
|  |  |  |  |  |  |  |  |  |
| **Side** | **Anatomical region** | **cluster size** | **x** | **y** | **z** | **Z** | **p value** | ***Reference*** |
| L | Middle temporal gyrus | 236 | -60 | -34 | -10 | 6.79 | <0.001* |  |
| L | Inferior frontal gyrus | 1055 | -48 | 26 | -10 | 6.74 | <0.001* |  |
| L | Superior frontal gyrus | 740 | -6 | 20 | 62 | 6.55 | <0.001* |  |
| R | Caudate nucleus | 103 | 12 | 12 | -2 | 6.37 | <0.001* |  |
| L | Medial frontal gyrus | 773 | -4 | 56 | 14 | 6.01 | <0.001* |  |
| L | Anterior cingulate gyrus |  | -6 | 40 | 26 | 5.89 | <0.001* |  |
| L | Medial orbital frontal gyrus |  | -8 | 58 | -12 | 5.49 | 0.001* |  |
| L | Inferior parietal lobule | 780 | -42 | -70 | 38 | 5.77 | <0.001* |  |
| L | Angular gyrus |  | -54 | -64 | 28 | 5.58 | 0.001* |  |
| L | Middle frontal gyrus | 111 | -40 | 8 | 52 | 5.57 | 0.001* |  |
| L | Supplementary motor area |  | -34 | 4 | 62 | 4.99 | 0.009* |  |
| L | Superior temporal gyrus | 39 | -40 | 20 | -30 | 5.36 | 0.002* |  |
| L | Temporal pole |  | -46 | 12 | -28 | 5 | 0.009* |  |
| L | Caudate nucleus | 77 | -10 | 16 | 0 | 5.29 | 0.002* |  |
| L | Retrosplenial cortex | 128 | -10 | -62 | 10 | 5.32 | 0.002* |  |
| L | Insula | 98 | -32 | 12 | -6 | 5.16 | 0.004* |  |
| R | Insula | 14 | 30 | 16 | -18 | 5.04 | 0.008* |  |
| R | Medial frontal gyrus | 19 | 2 | 58 | 16 | 5.02 | 0.009* |  |
| L | Thalamus | 10 | -4 | -10 | 8 | 4.97 | 0.011* |  |
| L | Ventromedial prefrontal cortex | 272 | -2 | 42 | -10 | 4.31 | 0.001** | *[39]* |
| L | Hippocampus | 225 | -24 | -20 | -16 | 3.55 | 0.017** | *[33]* |
