# Supplementary material for: The Hippocampus Remains Activated over the Long Term for the Retrieval of Truly Episodic Memories
Source: PLoS One. 2012 Aug 24;7(8):e43495. doi: 10.1371/journal.pone.0043495 (PMC3427359; doi:10.1371/journal.pone.0043495)
Supplement: Table S2 — Brain areas associated to the retrieval of initially episodic, later semantic memories (RK responses) compared to correct rejections at the 3-day delay. X, y, z refer to coordinates (in mm) in the Montreal Neurological Institute space. All regions listed are statistically significant at p<0.05 (FWE corrected, *) or psvc<0.05 (**), after correction in a small spherical volume (10 mm) around coordinates previously reported in the literature (specified in the last column). For brevity, each region is listed only once; when several peaks were observed in the same region, the coordinates refer to the strongest activation. Minimum cluster size: 10 contiguous voxels. (DOC) [file pone.0043495.s002.doc]

| **Table S2: Brain areas associated to the retrieval of initially episodic, later semantic memories (RK responses) compared to correct rejections at the 3-day delay.** | | | | | | | | |
| --- | --- | --- | --- | --- | --- | --- | --- | --- |
|
|  |  |  |  |  |  |  |  |  |
| **Side** | **Anatomical region** | **cluster size** | **x** | **y** | **z** | **Z** | **p value** | ***Reference*** |
| L | Inferior frontal gyrus | 1520 | -52 | 22 | 30 | 6.95 | <0.001* |  |
| L | Inferior orbital frontal gyrus |  | -50 | 36 | -2 | 6.62 | <0.001* |  |
| L | Medial frontal gyrus | 998 | -10 | 66 | 10 | 6.73 | <0.001* |  |
| L | Supplementary motor area |  | -6 | 8 | 66 | 6.72 | <0.001* |  |
| L | Middle frontal gyrus |  | -30 | 56 | 14 | 5.21 | 0.003* |  |
| L | Anterior cingulate gyrus | 227 | -6 | 36 | 28 | 6.58 | <0.001* |  |
| L | Inferior parietal lobule | 953 | -36 | -60 | 48 | 5.84 | <0.001* |  |
| L | Middle temporal gyrus | 246 | -60 | -34 | -10 | 5.65 | <0.001* |  |
| L | Precuneus | 172 | -6 | -64 | 30 | 5.55 | 0.001* |  |
| L | Cuneus |  | -6 | -74 | 28 | 4.8 | 0.023* |  |
| L | Precentral gyrus | 30 | -50 | -16 | 50 | 5.54 | 0.001* |  |
| L | Retrosplenial cortex | 153 | -6 | -52 | 8 | 5.29 | 0.002* |  |
| R | Retrosplenial cortex | 57 | 8 | -46 | 8 | 5.15 | 0.005* |  |
| L | Caudate nucleus | 19 | -10 | 16 | 4 | 5.07 | 0.007* |  |
| L | Posterior cingulate gyrus | 12 | -4 | -40 | 38 | 4.88 | 0.016* |  |
| L | Hippocampus | 247 | -28 | -34 | -10 | 3.84 | 0.007** | *[33]* |
| L | Ventromedial prefrontal cortex | 112 | -4 | 40 | -6 | 3.34 | 0.033** | *[39]* |
