# Supplementary material for: The Hippocampus Remains Activated over the Long Term for the Retrieval of Truly Episodic Memories
Source: PLoS One. 2012 Aug 24;7(8):e43495. doi: 10.1371/journal.pone.0043495 (PMC3427359; doi:10.1371/journal.pone.0043495)
Supplement: Table S3 — Brain areas associated to the retrieval of consistently episodic memories (RR responses) compared to correct rejections at the 3-month delay. X, y, z refer to coordinates (in mm) in the Montreal Neurological Institute space. All regions listed are statistically significant at p<0.05 (FWE corrected, *) or psvc<0.05 (**), after correction in a small spherical volume (10 mm) around coordinates previously reported in the literature (specified in the last column). For brevity, each region is listed only once; when several peaks were observed in the same region, the coordinates refer to the strongest activation. Minimum cluster size: 10 contiguous voxels. (DOC) [file pone.0043495.s003.doc]

| **Table S3: Brain areas associated to the retrieval of consistently episodic memories (RR responses) compared to correct rejections at the 3-month delay.** | | | | | | | | |
| --- | --- | --- | --- | --- | --- | --- | --- | --- |
|
|  |  |  |  |  |  |  |  |  |
| **Side** | **Anatomical region** | **cluster size** | **x** | **y** | **z** | **Z** | **p value** | ***Reference*** |
| L | Superior frontal gyrus | 6735 | -6 | 8 | 66 | Inf | <0.001* |  |
| L | Inferior orbital frontal gyrus |  | -48 | 26 | -10 | Inf | <0.001* |  |
| L | Inferior parietal lobule | 1636 | -42 | -60 | 40 | 7.3 | <0.001* |  |
| L | Angular gyrus |  | -50 | -62 | 28 | 7.12 | <0.001* |  |
| L | Middle temporal gyrus | 596 | -56 | -38 | -4 | 6.84 | <0.001* |  |
| R | Medial frontal gyrus | 1004 | 2 | 56 | 10 | 6.6 | <0.001* |  |
| R | Anterior cingulate gyrus |  | 2 | 46 | 20 | 6.19 | <0.001* |  |
| R | Supplementary motor area | 212 | 10 | 16 | 64 | 6.55 | <0.001* |  |
| L | Retrosplenial cortex | 1228 | -6 | -58 | 8 | 6.44 | <0.001* |  |
| L | Precuneus |  | -4 | -62 | 28 | 6.41 | <0.001* |  |
| L | Posterior cingulate gyrus |  | -6 | -54 | 28 | 6.21 | <0.001* |  |
| R | Caudate nucleus | 838 | 12 | 6 | 16 | 6.16 | <0.001* |  |
| L | Thalamus |  | -4 | -10 | 10 | 6.09 | <0.001* |  |
| L | Caudate nucleus |  | -8 | 16 | 2 | 6 | <0.001* |  |
| R | Inferior orbital frontal gyrus | 124 | 54 | 32 | -6 | 6 | <0.001* |  |
| L | Temporal pole | 178 | -46 | 14 | -30 | 5.92 | <0.001* |  |
| R | Cerebellum | 93 | 40 | -62 | -26 | 5.9 | <0.001* |  |
|  | Middle cingulate gyrus | 74 | 0 | -16 | 34 | 5.66 | <0.001* |  |
| L | Ventromedial prefrontal cortex | 237 | -4 | 40 | -10 | 5.48 | <0.001** | *[59]* |
| R | Insula | 36 | 30 | 14 | -18 | 5.43 | 0.001* |  |
| R | Lingual gyrus | 83 | 10 | -56 | 10 | 5.13 | 0.005* |  |
| R | Middle temporal gyrus | 18 | 56 | -2 | -30 | 5.11 | 0.006* |  |
| L | Hippocampus | 14 | -24 | -28 | -12 | 5.02 | 0.009* |  |
| R | Temporal pole | 17 | 44 | 18 | -30 | 4.96 | 0.011* |  |
| L | Inferior temporal gyrus | 37 | -58 | -2 | -26 | 4.94 | 0.012* |  |
