# Supplementary material for: The Hippocampus Remains Activated over the Long Term for the Retrieval of Truly Episodic Memories
Source: PLoS One. 2012 Aug 24;7(8):e43495. doi: 10.1371/journal.pone.0043495 (PMC3427359; doi:10.1371/journal.pone.0043495)
Supplement: Table S4 — Brain areas associated to the retrieval of initially episodic, then semantic memories (RK responses) compared to correct rejections at the 3-month delay. X, y, z refer to coordinates (in mm) in the Montreal Neurological Institute space. All regions listed are statistically significant at p<0.05 (FWE corrected, *) or psvc<0.05 (**), after correction in a small spherical volume (10 mm) around coordinates previously reported in the literature (specified in the last column). For brevity, each region is listed only once; when several peaks were observed in the same region, the coordinates refer to the strongest activation. Minimum cluster size: 10 contiguous voxels. (DOC) [file pone.0043495.s004.doc]

| **Table S4: Brain areas associated to the retrieval of initially episodic, then semantic memories (RK responses) compared to correct rejections at the 3-month delay.** | | | | | | | | |
| --- | --- | --- | --- | --- | --- | --- | --- | --- |
|
|  |  |  |  |  |  |  |  |  |
| **Side** | **Anatomical region** | **cluster size** | **x** | **y** | **z** | **Z** | **p value** | ***Reference*** |
| L | Inferior frontal gyrus | 1788 | -52 | 20 | 32 | 7.17 | <0.001* |  |
| L | Insula |  | -30 | 24 | -6 | 6.15 | <0.001* |  |
| L | Middle frontal gyrus |  | -46 | 6 | 46 | 5.82 | <0.001* |  |
| L | Supplementary motor area | 914 | -6 | 20 | 56 | 7.13 | <0.001* |  |
| L | Medial frontal gyrus |  | -4 | 30 | 40 | 6.91 | <0.001* |  |
| L | Inferior parietal lobule | 831 | -36 | -60 | 50 | 6.55 | <0.001* |  |
| L | Precuneus | 303 | -8 | -72 | 48 | 6.22 | <0.001* |  |
| L | Posterior cingulate gyrus | 139 | -2 | -18 | 32 | 6.18 | <0.001* |  |
| R | Medial frontal gyrus | 353 | 2 | 30 | 40 | 6.06 | <0.001* |  |
| R | Superior frontal gyrus |  | 4 | 20 | 46 | 5.47 | 0.001* |  |
| R | Supplementary motor area |  | 4 | 20 | 58 | 5.47 | 0.001* |  |
| R | Inferior parietal lobule | 45 | 48 | -38 | 52 | 5.28 | 0.002* |  |
| L | Middle temporal gyrus | 102 | -58 | -40 | -4 | 5.25 | 0.003* |  |
| L | Caudate nucleus | 40 | -8 | 16 | 2 | 5.15 | 0.005* |  |
| L | Superior orbital frontal gyrus | 23 | -24 | 56 | 0 | 5.04 | 0.008* |  |
| L | Superior frontal gyrus |  | -24 | 54 | 10 | 4.8 | 0.023* |  |
| R | Precuneus | 13 | 8 | -70 | 50 | 4.95 | 0.011* |  |
| R | Anterior cingulate gyrus | 12 | 2 | 44 | 20 | 4.9 | 0.014* |  |
| R | Postcentral gyrus | 45 | 52 | -32 | 48 | 4.73 | 0.03* |  |
| L | Angular gyrus | 105 | -44 | -60 | 36 | 4.49 | 0.001** | *[61]* |
| R | Superior parietal lobule | 291 | 34 | -66 | 48 | 4.58 | <0.001** | *[61]* |
|  | Retrosplenial cortex | 155 | 0 | -42 | 24 | 3.63 | 0.014 ** | *[62]* |
