# Supplementary material for: The Hippocampus Remains Activated over the Long Term for the Retrieval of Truly Episodic Memories
Source: PLoS One. 2012 Aug 24;7(8):e43495. doi: 10.1371/journal.pone.0043495 (PMC3427359; doi:10.1371/journal.pone.0043495)
Supplement: Table S5 — Brain areas in which activity during the retrieval of initially episodic, then semantic memories (RK responses) compared to correct rejections is higher after 3 days than 3 months, and conversely. X, y, z refer to coordinates (in mm) in the Montreal Neurological Institute space. All regions listed are statistically significant at p<0.05 (FWE corrected, *) or psvc<0.05 (**), after correction in a small spherical volume (10 mm) around coordinates previously reported in the literature (specified in the last column). For brevity, each region is listed only once; when several peaks were observed in the same region, the coordinates refer to the strongest activation. Minimum cluster size: 10 contiguous voxels. (DOC) [file pone.0043495.s005.doc]

| **Table S5: Brain areas in which activity during the retrieval of initially episodic, then semantic memories (RK responses) compared to correct rejections is higher after 3 days than 3 months, and conversely.** | | | | | | | | | |
| --- | --- | --- | --- | --- | --- | --- | --- | --- | --- |
|
|
|  |  |  |  |  |  |  |  |  |  |
|  | **Side** | **Anatomical region** | **cluster size** | **x** | **y** | **z** | **Z** | **p_svc_** | ***Reference*** |
| ***3 days > 3months*** | | |  |  |  |  |  |  |  |
|  | L | Hippocampus | 198 | -28 | -16 | -16 | 4.48 | <0.001 | *[63]* |
|  | R | Precentral gyrus | 269 | 48 | -14 | 46 | 4.26 | 0.002 | *[63]* |
|  | R | Fusiform gyrus | 282 | 40 | -52 | -20 | 3.53 | 0.019 | *[63]* |
| ***3 months > 3 days*** | | |  |  |  |  |  |  |  |
|  |  |  |  |  | *No suprathreshold clusters* | | | |  |
